# Exploring Cancer Cell Behavior In Vitro in Three-Dimensional Multicellular Bioprintable Collagen-Based Hydrogels

**DOI:** 10.3390/cancers11020180

**Published:** 2019-02-05

**Authors:** Daniela F. Duarte Campos, Andrea Bonnin Marquez, Cathal O’Seanain, Horst Fischer, Andreas Blaeser, Michael Vogt, Diana Corallo, Sanja Aveic

**Affiliations:** 1 Department of Dental Materials and Biomaterials Research, RWTH Aachen University Hospital, 52074 Aachen, Germany; andrea.bonnin@rwth-aachen.de (A.B.M.); c.oseanain@gmail.com (C.O.); hfischer@ukaachen.de (H.F.); 2 Medical Textiles and Biofabrication, Institut fuer Textiltechnik, RWTH Aachen University, 52074 Aachen, Germany; Andreas.Blaeser@ita.rwth-aachen.de; 3 Interdisciplinary Center for Clinical Research, RWTH Aachen University Hospital, 52074 Aachen, Germany; mvogt@ukaachen.de; 4 Fondazione Instituto di Ricerca Pediatrica Citta’ della Speranza, Neuroblastoma Laboratory, 35127 Padua, Italy; d.corallo@irpcds.org (D.C.); s.aveic@irpcds.org (S.A.)

**Keywords:** cancer cell, in vitro model, hydrogel, 3D, bioprinting

## Abstract

In vitro cancer 3D models are valuable tools to provide mechanistic insight into solid tumor growth, invasion, and drug delivery. The 3D spheroid model of solid tumors has been the most popular cancer model in use until now. However, previous studies have shown that these spheroid models lack sufficient morphological parameters, which may affect their response to chemicals. In this work, we proposed the fabrication of miniaturized 3D cancer models using collagen type I-based bioprintable bioinks. In the context of a mimicking model for advanced neuroblastoma studies, we showed that cancer cells contained in bioprintable bioinks formed Homer Wright-like rosettes, maintained their proliferative capacities and produced an equivalent Vimentin-rich matrix unlike that of non-bioprintable bioinks which made for poorer models. In addition, bioprintable bioinks were successfully bioprinted as compartmentalized 3D models in the centimeter scale, which was not feasible using non-bioprintable bioinks. In contrast to non-bioprintable hydrogels, we did not observe contraction in their bioprintable counterparts, which is an advantage for prospective 3D bioprinted models that should attain stable rheological and mechanical properties after bioprinting. By adopting this proposed system for the use of patient-derived primary tumor cells, the approach could be introduced as a first line strategy in precision medicine for testing the response of neuroblastoma cells to drugs, especially when disease progresses rapidly or patients do not respond to actual therapy regimens.

## 1. Introduction

In vitro cancer 3D models are valuable tools to provide mechanistic insight into solid tumor growth, invasion and drug delivery. They are slowly replacing the use of conventional 2D in vitro cell culture as the main method of adherent cell growth that has been practiced for decades in determining complex cellular and biological processes that occur during tumorigenesis. Advances in 3D cell culture technologies, biomaterials science, and biofabrication have enabled improvements in the development of in vitro cancer models. In recent years, spheroid-based models, hybrid models, and tumor–microvessel models have been studied and characterized [1]. The 3D spheroid model has hitherto been the most commonly used type of cancer models for solid tumors [2,3,4,5,6,7]. The advantage of using these spheroid models, for personalized drug screening for example, is that they allow a more reliable first line evaluation of the effects that chemotherapeutic agents have on patient’s autologous cancer cells than their 2D grown counterparts. However, previous studies have shown that these spheroid models lack sufficient morphological parameters, such as extracellular matrix components, which may affect their response to chemicals [2]. In order to recreate the tumor microenvironment, researchers have created tumor–microvessel models which incorporate a microvessel into an extracellular environment consisting of a hydrogel-like fibrin or collagen type I [8,9]. This type of approach is used to study interactions between cancer cells, their microenvironment and the tumor vasculature.

Neuroblastoma is the most common extracranial malignancy that occurs in preschool children, although occasionally it can be diagnosed in adolescents and adults [10]. Upon diagnosis, neuroblastoma can manifest as a localized or metastatic disease [11], hence determining the therapy regimen for the patient. Primary neuroblastoma tumors can be diagnosed at several sites including the adrenal glands, thorax, retroperitoneum, and pelvis. Metastatic sites are also numerous and include bone marrow, bones, lymph nodes, skin, liver, lung, and sometimes the central nervous system [12]. Today, treatment of patients with neuroblastoma classified within the high-risk group is very demanding and includes resection of the primary tumor mass, use of high-dose chemotherapy and autologous transplantation [13]. Nevertheless, the efficacy of currently proposed therapeutic options for high-risk patients with neuroblastoma is rather low. Among the possible reasons for the low efficacy of adopted therapies is lack of a complete knowledge about neuroblastoma onset, the biological processes that lay behind disease progression and the mechanisms that lead to drug resistance phenomenon. A significant barrier to the elucidation of the neuroblastoma pathophysiology is the lack of tumor material available for molecular and biochemical analysis post-diagnosis [14]. Therefore, a mimetic in vitro model of neuroblastoma could bridge the gap in overcoming these difficulties allowing the investigation of complex biological processes and the testing of new therapies for patients with neuroblastoma, particularly those belonging to the high-risk group. In addition, it would lead to a reduced use of animal models for the creation of patient-derived xenografts models.

With the advance of biofabrication strategies, in vitro 3D organotypic constructs have been created for a myriad of applications, ranging from bone and cartilage substitutes to cancer models [15,16]. In this work, we foster the fabrication of miniaturized 3D cancer models using collagen type I-based bioprintable bioinks. Collagen is a natural protein that constitutes a significant part of our tissues and it is as well abundant in neuroblastoma tumors. Therefore, the incorporation of collagen type I in in vitro cancer 3D models is an important aspect that has been considered in this study. In an ideally envisioned 3D model, cancer cells are printed as aggregates mimicking rosettes formed in native tumors, which are present in certain stromal cancers like neuroblastoma [17,18]. The surrounding of the cancer rosettes is printed with human mesenchymal stromal cells, interspaced with a mimicking vasculature. A proof-of-principle cell-free hydrogel-based 3D model was fabricated by drop-on-demand bioprinting and analysis of cancer cell interaction with stromal and endothelial cells was assessed in the form of 3D matrices. In this study, we compared cell interactions in the form of co- and tricultures in a non-bioprintable biological hydrogel (type I collagen) with a bioprintable bioink (agarose-type I collagen) as a potential microenvironment for cancer modeling using bioprinting.

## 2. Experimental

### 2.1. Preparation of Hydrogels

Agarose and collagen type I stock solutions were prepared as previously described [16,19]. Briefly, a stock solution of agarose (low gelling temperature, Sigma, Hamburg, Germany) of 3% (weight/volume) was prepared by dissolving the agarose powder in deionized water and autoclaving at 121 °C for 15 min. Collagen type I hydrogels (Biochrom, Berlin, Germany) were prepared by mixing eight parts of 0.4% acidic collagen solution with one part 10× DMEM (Biochrom) and with one part cell suspension in the corresponding medium (different for single cultures, co-cultures, and tricultures). Pure collagen type I hydrogels were not bioprinted. Bioprintable bioinks as in vitro 3D environments for cancer modeling were obtained by mixing 3% agarose with 0.3% collagen type I with final concentrations of 0.5% and 0.2%, respectively, for agarose (0.5% AG) and collagen (0.2% COL).

### 2.2. 5-Step Oscillatory Rheological Analysis in Liquid and Gelled Form

Oscillatory tests were performed in a rotational rheometer (Kinexus Pro+, Malvern Instruments, UK) using a 4° rotational cone-plate (PL6550905 SS, Malvern Instruments). Non-bioprintable (0.3% COL) and bioprintable bioinks (0.5% AG-0.2% COL) were measured both in liquid and in gelled form. Rheological analysis of liquid gels was performed at 37 °C and polymerized gels were analyzed at 25 °C after gelation of the hydrogels. A 5-step oscillatory sequence was performed for each of the hydrogels in both liquid and gelled form, based on previously published protocols [20]: (1) fixed step at low frequency (0.5 Hz) and with gentle oscillation at 0.5% strain; (2) frequency sweep between 0.5 and 50 Hz with fixed strain at 0.5%; (3) fixed step at low frequency (0.5 Hz) and 0.5% strain; (4) amplitude sweep with varying strain between 0.1 and 50% and fixed frequency at 0.5 Hz; and (5) fixed step at low frequency (0.5 Hz) and 0.5% strain. Steps 3 and 5 measured the recovery of the internal structure immediately after steps 2 and 4, respectively. The storage (G’) and loss (G’’) moduli were recorded over time (s) and plotted as point graphs.

### 2.3. Cell Isolation and Culture

In this work, three different cell types were used as single, co-, and tricultures: (1) human primary umbilical cord-derived mesenchymal stromal cells (UC-MSC, referred to as MSC), (2) human primary umbilical vein endothelial cells (HUVEC), and (3) human bone marrow-derived epithelial-neuroblastoma immortalized cells (SH-SY5Y). Human primary MSC and HUVEC were isolated according to previously published protocols [21,22]. MSC and HUVEC were isolated from umbilical cords provided by the Department of Gynecology and Perinatal Medicine (RWTH Aachen University Hospital) as approved by the local ethics committee (EK 218/14). Briefly, umbilical cords were cut in 1–2 cm pieces and opened lengthwise. The two veins and artery were removed in order to avoid the presence of blood cells and endothelial cells during the isolation of MSC from the Wharton’s jelly. The matrix of the Wharton’s jelly was scrapped and collected in a 344 U/mL collagenase solution (Worthington Biochemical Corporation, Lakewood, NJ, USA) at 37 °C overnight. On the next day, this suspension was centrifuged at 1200 rpm for 5 min and the supernatant was discarded. The pellet was resuspended in 2.5% trypsin (Gibco, Darmstadt, Germany) for 30 min and the reaction was stopped with medium (Mesenpan, PAN Biotech, Aidenbach, Germany). After another centrifugation step for 10 min, MSC were resuspended in medium and seeded in T25 cell culture flasks. Medium was changed every other day. The veins collected from the umbilical cord were used to isolate HUVEC. The veins were washed and flushed with phosphate buffered saline (PBS) in order to remove any remaining blood clog. The veins were flushed with 400 U/mL collagenase solution and incubated in a humidified atmosphere at 37 °C for 30 min. HUVEC were collected from the veins by flushing them with PBS. This solution was collected in a 50 mL tube and centrifuged at 500 rpm for 5 min. The supernatant was removed and the pellet was resuspended in EGM-2 medium (Lonza, Cologne, Germany) for further cultivation in 2% gelatin-coated (Sigma-Aldrich, Darmstadt, Germany) cell culture flasks. Medium was changed every other day. Immortalized neuroblastoma cell line SH-SY5Y was purchased from Deutsche Sammlung von Mikroorganismen und Zellkulturen (DSMZ) and cultured in DMEM (PAN Biotech) supplemented with 10% FCS and 1% penicillin/streptomycin/glutamine. Cells were transfected with Effectene transfection reagent (Qiagen, Milan, Italy) using the plasmid vector carrying the sequence for enhanced green fluorescent protein (GFP; 1 µg) and puromycin resistance. Selection of GFP-expressing cells was then achieved by culturing the cells under puromycin (0.5 µg/µL) determined selective growth conditions for 3 weeks. After selection, cells were checked for the maintenance of the *ALK* gene mutation F1174L, specific for SH-SY5Y cells (Supplementary Figure S1), by PCR amplification of the specific DNA region using the primer set

ALK Fwd
5′-GCAAGATTCTGGGTTTAGGC-3′
ALK Rvs
5′-CCATCGAGGAACTTGCTACC-3′

and subsequent Sanger sequencing as described elsewhere [23].

### 2.4. Preparation of Cell-Loaded Hydrogels as 3D Environments

Single, co-, and tricultures using MSC, HUVEC, and SH-SY5Y were prepared using bioprintable and non-bioprintable hydrogels. For non-bioprintable hydrogels, we used collagen type I hydrogel matrices. For bioprintable hydrogels, we used agarose-collagen type I blends. Final cell concentrations of single, co-, and tricultures were the same for both bioprintable and non-bioprintable hydrogels:

- MSC were loaded at 10^6^ cells per mL
- HUVEC were loaded at 3 × 10^6^ cells per mL
- SH-SY5Y were loaded at 10^6^ cells per mL

Cell number was determined using trypan-blue exclusion assay and Countess™ automated cell counter (Invitrogen, Darmstadt, Germany). Single cell cultures of MSC, HUVEC and SH-SY5Y were used as control cultures for the three cell types. In these samples cells were cultivated in their respective media (MSC were cultured in Mesenpan, HUVEC were cultured in EBM-2 and SH-SY5Y were cultured in DMEM). Single cultures in non-bioprintable hydrogels were prepared by mixing MSC, HUVEC, or SH-SY5Y in collagen type I hydrogel with a final concentration of 0.3%. Single cultures in bioprintable hydrogels were prepared by mixing the cells in agarose-collagen type I blends with a final concentration of 0.5% and 0.2%, respectively, for agarose and collagen. Cell-loaded hydrogels were casted with the respective cell densities for each cell type and polymerized at 37 °C for 30 min (non-bioprintable samples) or 1 min at 25 °C (bioprintable samples). Samples were incubated in their respective culture media at 37 °C for up to 14 days. One additional single culture of SH-SY5Y in EBM-2 was added to the experimental set-up for excluding possible differences with the single culture in DMEM.

Co-cultures of SH-SY5Y/MSC and SH-SY5Y/HUVEC were prepared with both non-bioprintable and bioprintable hydrogels and cultivated in endothelial medium (EGM-2). The final hydrogel concentrations and cell densities were the same as used for single cultures. The polymerization and culture conditions of co-cultures were the same as for single cultures, with the exception of the culture medium (EGM-2).

Tricultures of SH-SY5Y/MSC/HUVEC were prepared with both non-bioprintable and bioprintable hydrogels and cultivated in EBM-2. The final hydrogel concentrations and cell densities were the same as used for single and co-cultures. The polymerization and culture conditions of tricultures were the same as for co-cultures.

### 2.5. Macroscopic Evaluation of Cell-Loaded Hydrogel Models after In Vitro Culture

Macroscopic appearance and contraction of cell-loaded hydrogels after in vitro culture was recorded using a photographic camera (EF 100 mm, Canon, Tokyo, Japan).

### 2.6. Histological and Immunohistochemical Analysis

Cell-loaded hydrogel samples were histologically evaluated after in vitro culture. After 14 days of incubation, samples were fixed in 4% formaldehyde for 2 h, transferred to 70% ethanol, and dehydrated overnight. Then, samples were embedded in paraffin, cut into 8-µm slices, and stained histologically with hematoxylin and eosin (HE). Immunohistological staining was performed with Ki67 (1:200 dilution, DAKO, Santa Clara, CA, USA) and vimentin (1:250 dilution, Novus Biologicals, Littleton, CO, USA). Patients’ derived material was analyzed at the University Hospital of Padua, Italy. The tumor masses were examined under a light microscope (Nikon Eclipse TS 100, Southern Micro Instruments, Marietta, GA, USA) with a Nikon Coolpix camera under 10× and 20× magnification.

### 2.7. Two-Photon Imaging of Immunocytochemical Stainings

SH-SY5Y used in this study were stably expressing GFP. The imaging of SH-SY5Y distribution in the hydrogels was performed after DAPI co-staining (1:10000, Thermo Fisher). Samples were imaged with a two-photon laser scanning microscope (FV1000MPE, Olympus, Tokyo, Japan) mounted with a 25× NA 1.05 water dipping objective. Excitation wavelength was mode-locked to 800 nm using a pulsed Ti:Sapphire laser (Mai Tai DeepSee, Spectra Physics). Single images were recorded in the XY axis and collected at 1 µm z-stack intervals. In order to visualize human MSC and HUVEC, samples were stained after fixation in 4% formaldehyde with phalloidin-TRITC for labelling F-actin (1:50, Sigma-Aldrich) and DAPI for nuclear staining (1:10000, Thermo Fisher).

### 2.8. Proof-of-Principle Drop-on-Demand Printing of a Cell-Free 3D Cancer Model

**Figure 1:**
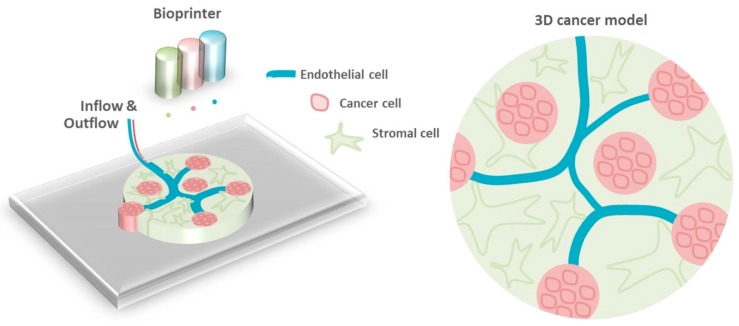
Schematic representation of the proof-of-principle experiment of a cell-free 3D cancer model. Cell-free 0.5% agarose (AG)-0.2% collagen (COL) bioprintable bioink mixed with three different colors was printed with architectures resembling that of the vasculature, Homer Wright rosettes and stromal matrix.

**Figure 2:**
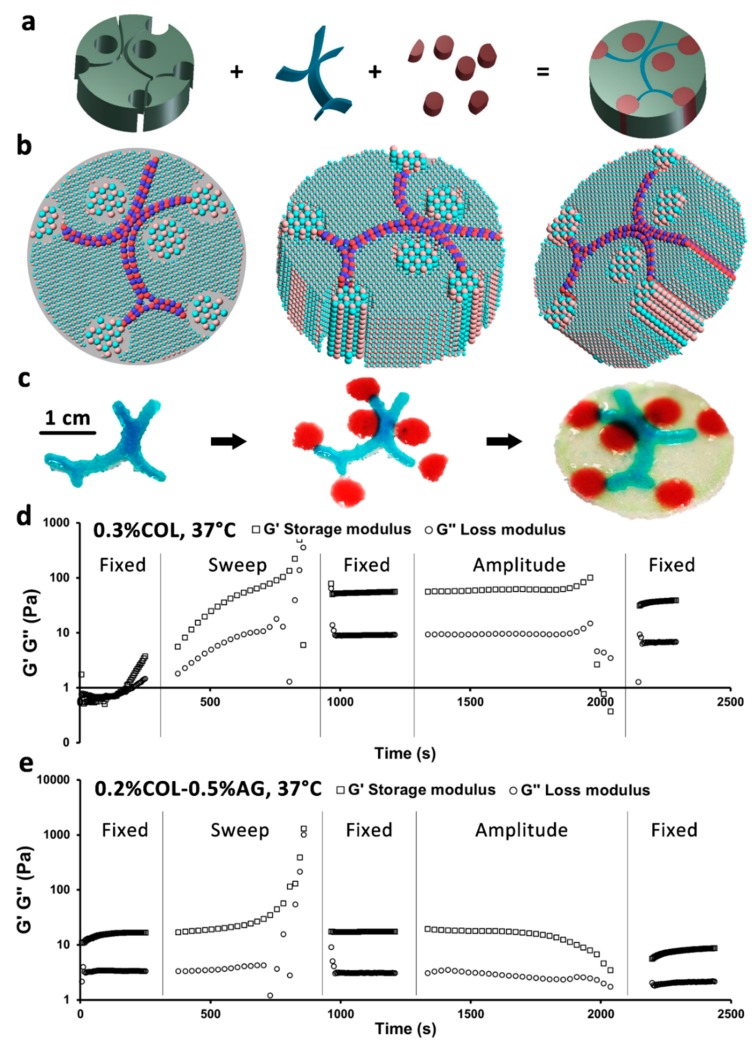
Printability study of bioinks and proof-of-concept printing of a 3D cancer model. (**a**) CAD design of 3D model, (**b**) its sliced version, and (**c**) macroscopic outcome after printing. Rheological measurement of (**d**) 0.3% COL and (**e**) 0.2% COL-0.5% AG with a 5-step oscillatory test. The nonrecovery of 0.3% COL structure after the frequency sweep test can be identified, in contrast to 0.2% COL-0.5% AG, which recovered its storage and loss moduli. G′ represents the storage modulus (squares) and G″ represents the loss modulus (circles).

Cell-free 0.5% AG-0.2% COL bioprintable bioink was printed with a resembling architecture as proposed in Figure 1, in order to show the potential of a drop-on-demand bioprinting strategy to create 3D miniaturized cancer models. The 3D model was designed in CAD (Autodesk Inventor 2016 vv.) and sliced using custom-made slicing software, as shown in Figure 2a,b. The bioink was colored with three different colors (blue, red, and green, Dr. Oetker, Bielefeld, Germany) mimicking the vasculature, cancer rosettes, and stroma, respectively. The three different colored bioinks were loaded in the bioprinting cartridges and a 3D model was printed drop-by-drop and layer-by layer. The bioprinter head was kept at 34 °C in order to keep the bioinks in the liquid state.

## 3. Results and Discussion

### 3.1. Blends of Agarose and Collagen Type I Hydrogels as BioprinTable 3D Environments

The creation of 3D cancer models using drop-on-demand bioprinting involves the use of hydrogels, which are not only biocompatible, but also succeed in mimicking in vitro microenvironments. In this work, we studied the printability of 0.2% COL-0.5% AG as bioinks for creating 3D models as well as the interaction of cancer cells with stromal and endothelial cells in non-bioprintable hydrogels as control matrices (0.3% COL) and in bioprintable hydrogels in light of their usability for bioprinting. A 3D model consisting of three distinct parts (vasculature, rosettes, and stroma) was designed in CAD and sliced (Figure 2a,b). Figure 2c shows the macroscopic result of the proof-of-principle printed 3D model. In order to predict the printability of the chosen hydrogel blend, we have rheologically characterized both non-bioprintable and bioprintable hydrogels (Figure 2d,e). Using a 5-step rheological test, we could show how small differences in oscillation can affect the storage and loss moduli of both studied materials, and how such rheological changes affect the hydrogel’s structural recovery. In the first step there is a noticeable and consistent increase of frequency (0.5 Hz) and strain (0.5%) in both storage and loss moduli of 0.3% COL. In contrast, 0.2% COL-0.5% AG bioprintable bioink revealed relatively constant parameters. After a frequency sweep from 0.5 to 50 Hz was applied to the gels, which induced a slight increase in both parameters for both studied hydrogels, 0.2% COL-0.5% AG displayed a recovery in structure (storage and loss moduli returned to initial values), whereas 0.3% COL maintained elevated storage and loss moduli (ten-fold higher than in step 1). There are two reasons why these findings can explain the printability of 0.2% COL-0.5% AG and not of 0.3% COL. Firstly, a ten-fold increase in the storage and loss moduli of 0.3% COL indicated a more solid-like behavior of the hydrogel [24]. During the bioprinting process, we deal with shear stresses which can affect not only cell viability but also the rheology of the bioink [25]. These rheological alterations have been simulated in our study with a frequency sweep test (step 2), which resulted in increased storage and loss moduli. Thus, these findings relate with an eventual gelation inside the printing cartridge (more solid-like behavior), which ultimately may result in clog formation in the printer head. Consequently, 0.3% COL was not suitable for bioprinting. Secondly, and in contrast to 0.3% COL, 0.2% COL-0.5% AG recovered its structure after the frequency sweep test. Thus, this was a successful indicator of good printability of the bioink, because it remained liquid.

### 3.2. Stromal Cells Produce Elastic Matrix with Contractive Properties

**Figure 3:**
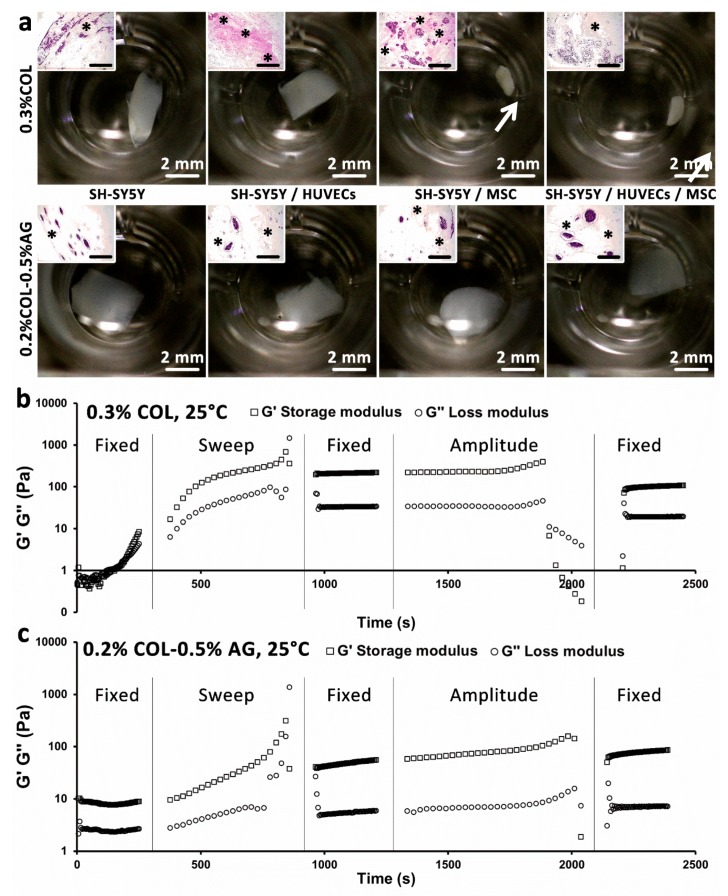
Rheological and elastic properties of 0.3% COL and 0.2% COL-0.5% AG gels and the effect of stromal cell elasticity on sample contraction. (**a**) Macroscopic images of both hydrogel cultures showing contraction of 0.3% COL only in the presence of MSC (inserts show histological HE stainings; * indicate the formation of elastic matrix; scale bars represent 200 µm). Rheological measurement of (**b**) 0.3% COL and (**c**) 0.2% COL-0.5% AG with a 5-step oscillatory test. A rapid increase in storage and loss moduli of 0.3% COL at low frequency (0.5 Hz) can be observed, in contrast to 0.2% COL-0.5% AG, which remained constant. G′ represents the storage modulus (squares) and G″ represents the loss modulus (circles).

Cancer tissue that is formed in the human body consists not only of cancer cells, but also of other cell types surrounding the tumor. Stromal cancers like neuroblastoma are characterized by the presence of stromal cells like Schwann cells and can be stroma-rich or stroma-poor [26]. Thus, in our study, we were interested in investigating the interplay between SH-SY5Y and human MSC as a native conditioning for the envisioned bioprinted model. We co-cultured both cell types in non-bioprintable hydrogels and bioprintable bioinks to predict their behavior when integrated in the 3D bioprinted model. In addition, we studied the supplementary culture with endothelial cells, also present in native cancers. Interestingly, the presence of human MSC in co- and tricultures with SH-SY5Y (and HUVEC) resulted in a macroscopically visible contraction of the non-bioprintable hydrogels (0.3% COL, Figure 3a, first row). In contrast, this was not observed in 0.3% COL gels without the MSC and in bioprintable hydrogels (Figure 3a, second row). To further investigate the reason behind the collagen hydrogel contraction, we performed a rheological characterization of both types of hydrogels in their gelled form (Figure 3b,c). During the first step of the oscillatory rheological test, it was shown that the storage and loss moduli of 0.3% COL rapidly increased when a low frequency (0.5 Hz) was applied. In contrast, both moduli remained relatively constant for the bioprintable bioink (0.2% COL-0.5% AG). This outcome can be explained by the unstable structural state of 0.3% COL when it is not combined with agarose. It also correlates with previous studies which indicated weak mechanical properties of pure collagen hydrogels [16,27]. Based on the mentioned rheological and mechanical properties of 0.3% COL, we concluded that the combination of this material with agarose, in order to create a bioprintable bioink (0.2% COL-0.5% AG), is a suitable solution for bioprinting 3D cancer models, which do not contract after in vitro cultivation. In addition, histological stainings showed evidence of elastic matrix production not only in non-bioprintable hydrogels but also in bioprintable bioinks (Figure 3a, inserts, HE staining).

### 3.3. Cancer Cells form Homer Wright-Like Rosettes in 3D Cancer Models Similar to that of Native Tissue

**Figure 4:**
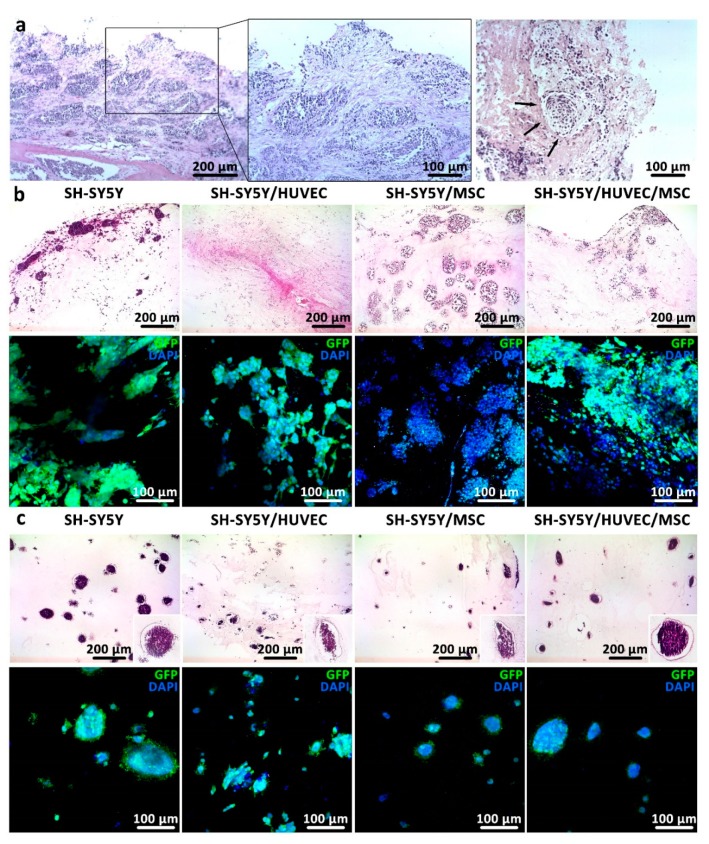
Cancer cells formed Homer Wright-like rosettes in bioprintable bioinks as they do in human neuroblastoma tissue. (**a**) Histological section of human neuroblastoma tumors from two high-risk patients were stained with HE. The tumor masses were examined under light microscope with 10× (first image on the left) and 20× (two images on the right) magnification. Arrows indicate Homer Wright-like rosettes structures. (**b**,**c**) Histological sections (first row) and immunocytochemical images (second row) of (**b**) 0.3% COL and (**c**) 0.2% COL-0.5% AG after 14 days of in vitro culture show organization of tumor and nontumor cells in non-bioprintable and bioprintable hydrogels, respectively.

As a proof-of-principle, the 3D cancer model proposed in this work should ideally resemble the microenvironment of native neuroblastoma cancer. It is well-known that neuroblastoma rosettes can be found in histological and aspirated bone marrow smears [28]. Neuroblastoma rosettes or Homer Wright rosettes are characterized by a halo of tumor cells surrounding a central region (Figure 4a) [29]. In this work, we showed evidence for rosette forming cancer cell colonies, both in non-bioprintable (0.3% COL, Figure 4b) and bioprintable bioinks (0.2% COL-0.5% AG, Figure 4c). Histological HE staining showed similar rosette formation as seen in native human neuroblastoma sections for both studied hydrogels. Qualitatively, the most similar rosette formation was observed in non-bioprintable hydrogels with co-cultures of SH-SY5Y and MSC. This was further shown by immunocytochemical images of GFP/DAPI stained cells obtained by two-photon microscopy in four different culture types where we can confirm the complexity of tumor formation in adopted 3D growing conditions. This is the first set of data showing that it is possible to recreate the microenvironment of neuroblastoma stromal cancer in in vitro matrices by the formation of Homer Wright-like rosettes, since past studies were limited to 2D or 3D spheroid cultures with insufficient evidence of such [2,30]. A study from Curtin and colleagues introduced a collagen-based scaffold as a neuroblastoma model with homogenous cell distribution [30]. However, the histological analysis presented in their study did not show evidence for tumor cells arranged as a halo surrounding a central region. Contrarily, in our study we not only showed cancer rosette formation in non-bioprintable hydrogels, but also very encouraging results in bioprintable hydrogels. Additionally, we did not observe any substantial difference in cell proliferation, rosette formation and hydrogel rheology between the samples grown in EGM-2 medium, or cell specific medium such as DMEM (SH-SY5Y) and Mesenpan (MSC), confirming that the adopted growth conditions were optimal for all three cell types used in co-cultures (data not shown).

### 3.4. Cancer Cells are Proliferative in 3D Cancer Models and Produce Vimentin-Rich Matrix

**Figure 5:**
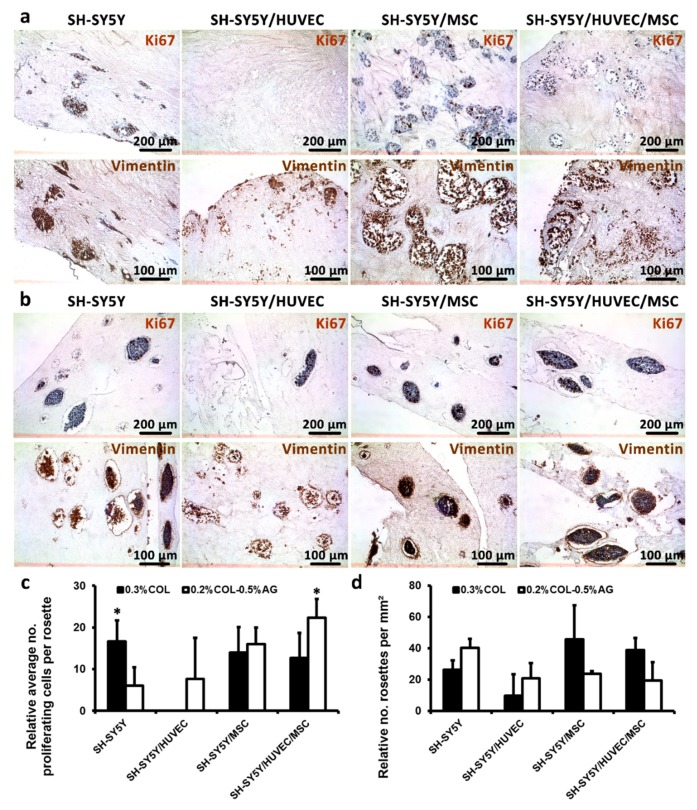
Analysis of cell proliferation, formation of rosettes in 3D hydrogels and expression of the mesenchymal marker vimentin. (**a**,**b**) First rows: cell proliferation was assessed by Ki67 marker confirming that the cells maintained their capacity to amplify even after 14 days of culture in both non-bioprintable (**a**) and bioprintable hydrogels (**b**). (**a**,**b**) Second rows: mesenchymal marker Vimentin, the expression of which correlates with the occurrence of tumorigenesis and increased metastatic capacity of tumor cells that was validated in mono- and co-cultures. In both types of hydrogels, we observed extensive positivity for this protein marker. (**c**) Relative quantification of average number of proliferating cells was measured for each rosette and presented as means ± SD. (**d**) Relative number of rosettes counted per mm² was calculated to evaluate possible differences between two types of hydrogels tested in our study. The highest number of formed rosettes was found in SH-SY5Y/MSC co-cultured in non-bioprintable hydrogels. Statistical analysis was performed with Student’s *t*-test and significance was defined as * *p* < 0.05.

Advanced neuroblastoma cancer cells are highly proliferative cells in vivo and in vitro [31]. Nevertheless, patient-derived tumor material available for preclinical and biological studies is often scarce making more comprehensive studies of tumor biology almost impossible. As a consequence, new knowledge about neuroblastoma pathophysiology is limited, making it difficult to propose more effective therapy options that could increase the current survival rate of high-risk patients that is still below 40% [32]. Thus, it is necessary to create biocompatible, long-term 3D growth conditions in which cells can recapitulate tumor specific organization. Ideally, these 3D tumoroid models cells should continue to proliferate and produce stromal-rich matrix as they do in native tumors. Our results showed that SH-SY5Y cultured both in non-bioprintable and bioprintable bioinks stained positive for the proliferative marker Ki67, as demonstrated by immunohistochemical staining after 14 days of growth (Figure 5a,b), confirming that the growth conditions adopted in this study did not interfere with the proliferative rate typical of SH-SY5Y in tumors. In addition, we analyzed our samples for the expression of Vimentin—an intermediate filament component which plays a role in the regulation of cell movement and is highly expressed by malignant cells [33]. We confirmed Vimentin-rich zones of tumor cells which are indicative of an aggressive phenotype. Interestingly, we could also observe some extracellular, punctuate-like distribution of Vimentin, a phenomenon that was described along with inflammation-related cell processes [34], but was not reported previously for neuroblastoma. The relative average number of proliferating cells per rosette in cancer monocultures in non-bioprintable bioinks was statistically higher than bioprintable hydrogels (Figure 5c). However, tricultures in bioprintable bioinks showed the opposite result, i.e., there were statistically more proliferating cells inside the rosettes formed in bioprintable bioinks relative to non-bioprintable bioinks. Moreover, the relative number of rosettes per mm² was equivalent for all single, co-, and tricultures and both non-bioprintable and bioprintable hydrogels (Figure 5d). This finding was relevant for the goal of our approach, since it showed that the presence of agarose in bioprintable bioink blends did not negatively affect the formation of Homer Wright-like rosettes. Therefore, the proposed bioprintable bioinks can be considered suitable for mimicking a native neuroblastoma tumor in vitro.

### 3.5. Analysis of Cancer Cell Distribution and Interaction in 3D In Vitro Models

**Figure 6:**
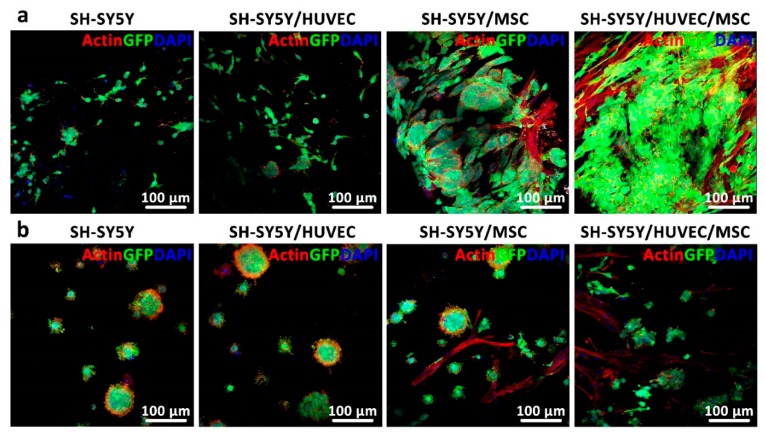
Two-photon images of SH-SY5Y distribution and interaction with HUVEC, MSC, and both HUVEC/MSC in non-bioprintable and bioprintable hydrogels. (**a**,**b**) Vascular tube formation was not observed in any of the studied bioinks in the presence of SH-SY5Y, contrarily to what was previously shown in the literature [21,22], whereas interaction of SH-SY5Y cells rosettes and MSC (elongated cells in red) was achieved.

The proposed theoretical 3D cancer model (Figure 1) was composed of three parts: the vasculature, rosettes, and stromal matrix. The obtained results for cell distribution within non-bioprintable and bioprintable hydrogels could justify the necessity for bioprinting a vasculature into the 3D model instead of random combination of endothelial cells with other cell types to form vascular tubes. This statement can be explained based on one major finding, which relates to unsuccessful vasculature formation in both studied matrices (Figure 6a,b). All three types of cells contained in the studied hydrogels were stained with phalloidin for the visualization of F-actin cytoskeleton and hence cell shape. In case vasculature was formed by HUVEC, it would have been microscopically visible (in red). A low-to-moderate level of Caspase 3 staining (Supplementary Figure S2) confirmed apoptosis induction in HUVEC cultures, which could have been the reason for the lack of vascularization. Previous studies have shown that the interaction of HUVEC and MSC is advantageous for the formation of vascular tubes, even inside bioprintable bioinks [21,22]. However, in this study we showed that such tubular structures did not arise in the presence of SH-SY5Y cells, at least not within 14 days of culture. This phenomenon is not surprising as it is known that the neoangiogenic process in tumors, newly formed microvessels display numerous abnormalities in their structure and function [35]. Moreover, it is likely that tissue-engineered models need to be adapted for application in the oncological field by allowing HUVECs to mature and form vascular tubes before introducing cancer cells to the model [36]. Thus, it is likely that a bioprinted vasculature consisting either only of HUVEC or a co-culture of HUVEC and MSC would be a more favorable option for the generation of artificial vessels that will be required for providing nutrients to cancer and stromal cells contained in the proposed 3D model. In fact, the interaction of neuroblastoma cells and stromal cells (MSC) was very intense and was clearly observed in both types of hydrogels (Figure 6a,b third column), indicating the requirement of stromal support for tumor cell growth.

## 4. Conclusions

In this work, we proposed the fabrication of miniaturized 3D cancer models using collagen type I-based bioprintable bioinks. In the context of a mimicking model for advanced neuroblastoma studies, we showed that cancer cells contained in bioprintable bioinks formed Homer Wright-like rosettes, maintained their proliferative capacities and produced Vimentin-rich matrices. In addition, the presence of MSC triggered the production of elastic matrix, which consequently forced non-bioprintable hydrogels to contract. In contrast, we could not observe contraction in bioprintable hydrogels, which is an advantage for prospective 3D bioprinted models that should attain stable rheological and mechanical properties after bioprinting. Moreover, vascular tube formation in the presence of cancer cells was not observable neither in non-bioprintable hydrogels nor in bioprintable bioinks. A possible explanation for this is the small size of the generated tumoroids which may offset the need for such structures given that nutrients can easily reach tumor cells by diffusion. On the contrary, it is known from the literature and has been confirmed in our lab that the 3D co-culture of HUVEC and MSC, in the absence of cancer cells, leads to a production of regular and well-defined microvasculature, implying a more cooperative dynamic between these two cell types. Thus, to avoid the described limitation met during the histological examination of our models, we proposed a novel design for 3D stromal tumoroids, in which the vasculature should contain only HUVEC, or a combination of HUVEC and MSC, in order to achieve a successful route for nutrients to reach the resting cells of the developed bioprintable model. In this manner, by adopting bioprinting methodology we could guarantee a uniform and more controlled way of generating the 3D tumoroids that can subsequently be used for cell biology, molecular or pharmaceutical studies in a median-throughput range. In addition, by adopting this system for the use of patient-derived primary tumor cells, the approach could be introduced as a first line therapy in a precision medicine approach to testing the response of neuroblastoma cells to drugs, especially when disease progresses rapidly, or patients do not respond to actual therapy regimens.

## Supplementary Materials

The following are available online at http://www.mdpi.com/2072-6694/11/2/180/s1, Figure S1: SH-SY5Y cells after 3 weeks of growth in puromycin selective conditions. (**a**) A green signal emanates from neuroblastoma cells expressing the enhanced green fluorescent protein (GFP). After 3 weeks in selective medium all cells were GFP positive and were transferred into the normal, nonselective DMEM medium. (**b**) Sanger sequencing confirmed the presence of the point mutation in SH-SY5Y cells at position 1174 of the *ALK* gene, the most frequent alteration described in ~8% of de novo neuroblastomas. Chromatogram indicates the heterozygosity for the specific *ALK* gene locus where both wild-type TTC sequence and mutated TTA sequence can be observed, leading to an amino acid change from phenylalanine (F) to Leucine (L); Figure S2: Micrographs of Caspase 3 immunohistochemical staining. The expression of Caspase 3 was examined in the sections of non-bioprintable (COL) and bioprintable (COL-AG) hydrogels of in vitro co-culture of tumor (SH-SY5Y) and nontumor (HUVEC and MSC) cells. Apoptotic (Caspase 3 positive) cells were recognized as dark brown colored marks. Nuclei were counterstained with hematoxylin. Negative control (NEG CTRL) was colored only with hematoxylin.
