# Supplementary material for: Exploring Cancer Cell Behavior In Vitro in Three-Dimensional Multicellular Bioprintable Collagen-Based Hydrogels
Source: Cancers (Basel). 2019 Feb 5;11(2):180. doi: 10.3390/cancers11020180 (PMC6406373; doi:10.3390/cancers11020180)
Supplement: Supplementary file 1 [file cancers-11-00180-s001.pdf]

cancers

Supplementary Materials: Exploring Cancer Cell Behavior In Vitro in Three-Dimensional Multicellular Bioprintable Collagen-Based Hydrogels

Daniela F. Duarte Campos, Andrea Bonnin Marquez, Cathal O'Seanain, Horst Fischer, Andreas Blaeser, Michael Vogt, Diana Corallo and Sanja Aveic

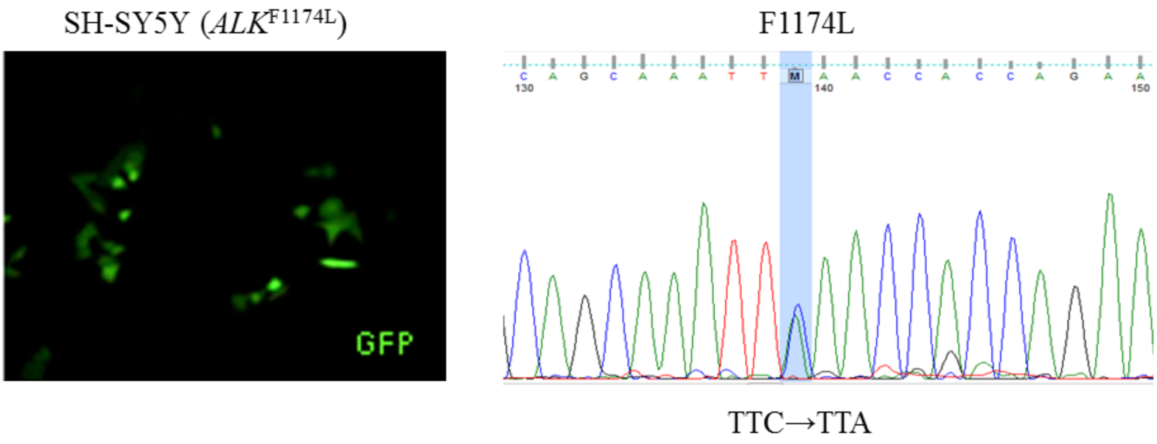

Figure S1. SH-SY5Y cells after 3 weeks of growth in puromycin selective conditions. (**a**) A green signal emanates from neuroblastoma cells expressing the enhanced green fluorescent protein (GFP). After 3 weeks in selective medium all cells were GFP positive and were transferred into the normal, non-selective DMEM medium. (**b**) Sanger sequencing confirmed the presence of the point mutation in SH-SY5Y cells at position 1174 of the *ALK* gene, the most frequent alteration described in around 8% of de novo neuroblastomas. Chromatogram indicates the heterozygosity for the specific *ALK* gene locus where both wild-type TTC sequence and mutated TTA sequence can be observed, leading to an amino acid change from phenylalanine (F) to Leucine (L).

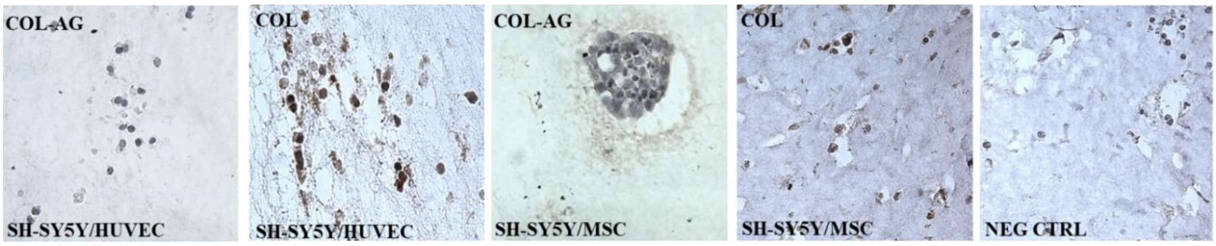

Figure S2. Micrographs of Caspase 3 immunohistochemical staining. The expression of Caspase 3 was examined in the sections of non-bioprintable (COL) and bioprintable (COL-AG) hydrogels of in vitro co-culture of tumor (SH-SY5Y) and non-tumor (HUVEC and MSC) cells. Apoptotic (Caspase 3 positive) cells were recognized as dark brown colored marks. Nuclei were counterstained with hematoxylin. Negative control (NEG CTRL) was colored only with hematoxylin.

© 2019 by the authors. Licensee MDPI, Basel, Switzerland. This article is an open access article distributed under the terms and conditions of the Creative Commons Attribution (CC BY) license (http://creativecommons.org/licenses/by/4.0/).
